# *FLT3* Mutations in Acute Myeloid Leukemia: Key Concepts and Emerging Controversies

**DOI:** 10.3389/fonc.2020.612880

**Published:** 2020-12-23

**Authors:** Vanessa E. Kennedy, Catherine C. Smith

**Affiliations:** Division of Hematology and Oncology, Department of Medicine, University of California San Francisco, San Francisco, CA, United States

**Keywords:** Acute Myeloid Leukemia, FLT3 inhibitor, FLT3 resistance, FLT3 inhibitor maintenance, non-canonical FLT3 mutation

## Abstract

The FLT3 receptor is overexpressed on the majority of acute myeloid leukemia (AML) blasts. Mutations in *FLT3* are the most common genetic alteration in AML, identified in approximately one third of newly diagnosed patients. *FLT3* internal tandem duplication mutations (*FLT3-*ITD) are associated with increased relapse and inferior overall survival. Multiple small molecule inhibitors of FLT3 signaling have been identified, two of which (midostaurin and gilteritinib) are currently approved in the United States, and many more of which are in clinical trials. Despite significant advances, resistance to FLT3 inhibitors through secondary *FLT3* mutations, upregulation of parallel pathways, and extracellular signaling remains an ongoing challenge. Novel therapeutic strategies to overcome resistance, including combining FLT3 inhibitors with other antileukemic agents, development of new FLT3 inhibitors, and FLT3-directed immunotherapy are in active clinical development. Multiple questions regarding *FLT3-*mutated AML remain. In this review, we highlight several of the current most intriguing controversies in the field including the role of FLT3 inhibitors in maintenance therapy, the role of hematopoietic cell transplantation in *FLT3-*mutated AML, use of FLT3 inhibitors in *FLT3* wild-type disease, significance of non-canonical *FLT3* mutations, and finally, emerging concerns regarding clonal evolution.

## FLT3 Epidemiology, Biology, and Prognostic Associations

Acute Myeloid Leukemia (AML) is an aggressive hematologic malignancy characterized by a heterogenous genetic landscape and complex clonal evolution (1). Fms-like tyrosine kinase 3 (FLT3), a member of the receptor tyrosine kinase family, is widely expressed in hematopoietic progenitor cells and is overexpressed on the majority of AML blasts (2). Upon binding to the FLT3 ligand, FLT3 receptors activate and dimerize, leading to conformational change, cellular proliferation, and inhibition of apoptosis and differentiation (3). Mutations in *FLT3* are the most common genomic alteration in AML, identified in approximately one-third of newly diagnosed adult patients (4), and are common in pediatric AML as well (5).

*FLT3* mutations can be subdivided into internal tandem duplicates (ITD), present in approximately 25% of patients, and point mutations in the tyrosine kinase domain (TKD), present in approximately 5%. Both *FLT3*-ITD and *FLT3-*TKD mutations are constitutively activating, leading to ligand-independent FLT3 signaling and cellular proliferation (3).

## *FLT3*-ITD and *FLT3*-TKD

*FLT3-*ITD mutations are in-frame duplications of variable size, ranging from 3 to >1,000 nucleotides, and are located within the receptor’s autoinhibitory juxatamembrane domain. In wild type (WT) *FLT3*, the FLT3 juxtamembrane domain inhibits receptor activation; the presence of ITDs disrupts this inhibitory effect, resulting in constitutive activation (6). Clinically, *FLT3-*ITD mutated AML is associated with higher rates of relapse and inferior overall survival, although the full prognostic impact is affected both by mutant allele burden and presence of co-existing mutations (7). High allele ratio (AR; *FLT3*-ITD^high^), generally defined as a *FLT3*-ITD to *FLT3*-WT ratio of ≥0.5, is associated with higher disease risk. Low AR (*FLT3*-ITD^low^) is associated with favorable risk in patients with a co-occurrent nucleophosmin 1 (*NPM1*) mutations and intermediate risk in patients with *NPM1*-WT. These associations are reflected in the 2017 European LeukemiaNet (ELN) risk stratification of AML (8).

*FLT3-*TKD mutations are point mutations within the receptor’s activation loop which stabilize the active kinase conformation and also result in constitutive kinase activation (3). In contrast with *FLT3-*ITD mutant AML, the prognostic relevance of *FLT3-*TKD mutations is less clear and may be dependent on the presence of co-occurring mutations and cytogenetic changes (9, 10). Currently, the presence of a *FLT3*-TKD mutation does not alter formal AML risk assessment (8).

## Testing Considerations

Given the prognostic and treatment implications, testing for *FLT3*-ITD in patients with newly diagnosed AML is recommended by both ELN and National Comprehensive Cancer Network (NCCN) guidelines (8, 11). In addition, given the clonal evolution observed in AML, *FLT3* mutations can be gained or lost at disease relapse and progression. In an individual patient, the presence of *FLT3* mutations will often need to be re-assessed over time.

Currently, there remains considerable variability in *FLT3* assay types, associated sensitivity and specificity, and turnaround time among treating centers (12). There are two main methods for determining *FLT3* status are polymerase chain reaction (PCR)-based assays and next generation sequencing (NGS). Due to competition from the *FLT3-*WT allele, the sensitivity of standard PCR-based assays may be lower than NGS-based methods, although this can be overcome using patient-specific PCR primers (13).

The *FLT3* allelic frequency (AF) has also been used to define *FLT3*-ITD positivity, primarily in research studies. Unlike the AR, which calculates the ratio of the area under the curve (AUC) of mutant to WT alleles (*FLT3-*ITD/*FLT3*-WT), the AF calculates the fraction of mutant alleles as a percentage of all *FLT3* alleles (*FLT3-*ITD/*FLT3*-WT + *FLT3*-ITD). For example, a *FLT3* AR of 0.5 (0.5 ITD/1.0 WT) would be equal to a *FLT3* AR of 0.33 (0.5 ITD/0.5 ITD + 1.0 WT = 0.33 AF). NGS studies are typically reported as VAF while PCR assays typically report AR.

Historically, *FLT3*-ITD has been inherently difficult to detect using NGS. NGS relies on the reconstruction of short (<300 base pair) sequences, and longer length ITDs may not be detected (14, 15) although this can be overcome using novel bioinformatic approaches (16). NGS is becoming more commonly used, especially in monitoring for *FLT3*+ minimal residual disease (MRD) following treatment. An NGS-based FLT3 assay is now commercially available and is currently used in in an ongoing trial of gilteritinib maintenance therapy following hematopoietic cell transplantation (HCT) (NCT02997202).

Both PCR and NGS-based assays are supported by the current NCCN guidelines (8); however, ELN risk stratification is dependent upon *FLT3* AR, which can only be determined by PCR. There is currently no standardized methodology or laboratory reference values for AR determination, and the current cut-off of ≥0.5 has not been prospectively validated. In retrospective analyses, higher AR is generally associated with inferior clinical outcomes (17), although these studies are prior to the widespread use of FLT3 inhibitors. It is likely that impact of AR on prognosis exists on a continuum, rather than a discrete cut-off. Finally, many patients do not receive *FLT3* testing at all. In a large survey by the American College of Pathologists in 2015, only 51% of new AML referrals received *FLT3* testing (18).

## Overview of Currently Established FLT3 Inhibitors

Given the prevalence and poor prognosis of *FLT3*-ITD mutated AML, targeting FLT3 signaling through small molecule inhibitors is a promising therapeutic strategy. FLT3 inhibitors can be stratified using two primary schema: first *vs* second generation FLT3 inhibitors and type I *vs* type II FLT3 inhibitors. FLT3 inhibitors currently in use or in active development, including toxicity profiles, are detailed in **Table 1**.

**Table 1:** Established FLT3 inhibitors and common toxicity profiles.

FLT3 Inhibitor	Type	Common or Notable Toxicities
**First Generation**		
Midostaurin	Type I	**Hematologic:** Cytopenias, including febrile neutropenia and abnormal bruising or bleeding; differentiation syndrome **Constitutional:** Pyrexia, flu-like symptoms **Cardiac:** Cardiac failure (rare, <5%) **GI:** Abdominal pain, nausea, vomiting, diarrhea, stomatitis, AST or ALT increase (19–21)
Sorafenib	Type II	**Hematologic:** Cytopenias, usually mild; differentiation syndrome **Constitutional:** Fatigue, can be severe in ~6% of patients **Cardiac:** Hypertension, cardiac ischemia (rare, <5%) **GI:** Diarrhea **Dermatologic:** Rash, erythema, hand-foot skin reaction (22–24)
**Second Generation**		
Quizartinib	Type II	**Hematologic:** Cytopenias, including febrile neutropenia and abnormal bruising or bleeding; differentiation syndrome **Cardiac:** QTc prolongation (dose-dependent, can be severe) **GI:** Abdominal pain, nausea, anorexia (25, 26)
Gilteritinib	Type I	**Hematologic:** Cytopenias, usually mild; differentiation syndrome **GI:** Diarrhea, pancreatitis (rare, <5%, but can be severe), AST or ALT increase **Neurologic:** Peripheral neuropathy, headache (27, 28)
Crenolanib	Type I	**Hematologic:** Differentiation syndrome **GI:** Abdominal pain, nausea, AST or ALT increase **Other:** Peripheral edema (29, 30)

Current usage and ongoing trials of established FLT3 inhibitors in newly diagnosed and relapsed/refractor (R/R) AML are summarized in **Tables 2** and **3**, respectively.

**Table 2:** Select Trials of Established FLT3 inhibitors in newly diagnosed AML.

NCT/Trial Identifier	Phase	Treatment Setting	Patient Population
**Midostaurin**			
NCT01477606/AMLSG 16-10	II	Combination with induction and consolidation chemotherapy, plus maintenance	*FLT3-*ITD; age 18−70
NCT03512197	III	Midostaurin *vs* placebo in combination with induction and consolidation chemotherapy	*FLT3-*WT, defined as *FLT3-*ITD, D835, or I836 AR < 0.05; age ≥ 18; no prior FLT3 inhibitor therapy
NCT03900949	I	Combination with induction chemotherapy plus gemtuzumab ozogamicin	*FLT3-*ITD or -TKD, CD33 +; age ≥ 18
NCT04385290/MOSAIC	II	Combination with induction chemotherapy plus gemtuzumab ozogamicin	*FLT3-*ITD or -TKD, age 18−75
**Gilteritinib**			
NCT02236013	I	Combination with induction and consolidation chemotherapy	All AML; age ≥ 18
NCT04027309/HOVON 156	III	Gilteritinib *vs* Midostaurin in combination with induction and consolidation chemotherapy, plus maintenance	*FLT3*-ITD or -TKD AML; age ≥ 18
**Quizartinib**			
NCT02668653/QuANTUM-First	III	Quizartinib *vs* Placebo in combination with induction and consolidation chemotherapy, plus maintenance post-chemotherapy and post-HCT	*FLT3*-ITD AML; age 18−75
NCT03723681	I	Combination with induction and consolidation chemotherapy	All AML; age 18−75
NCT03135054	II	Combination with Omacetaxine mepesuccinate	*FLT3*-ITD AML; age ≥ 18
NCT04047641	II	Combination with cladribine, idarubicin, and cytarabine	All AML, MDS; age 18–65 (first-line cohort)
NCT04107727	II	Quizartinib *vs* placebo in combination with induction and consolidation chemotherapy	*FLT3-*WT, defined as *FLT3-*IT AR < 0.03; age 18–70
NCT04128748	I/II	Combination with liposomal cytarabine and anthracycline (CPX-351; Vyxeos)	All AML; age ≥ 60
**Crenolanib**			
NCT03258931	III	Crenolanib *vs* Midostaurin in combination with induction and consolidation chemotherapy, plus maintenance	*FLT3-*ITD or -TKD, age 18−60

**Table 3:** Select Trials of Established FLT3 inhibitors in R/R AML.

NCT/Trial Identifier	Phase	Treatment Setting	Patient Population
**Quizartinib**			
NCT03989713/Q-HAM	II	Combination with salvage chemotherapy (Ara-C, Mitoxantrone); R/R after first-line treatment, including HCT	*FLT3*-ITD AML; age 18−75
NCT04047641	II	Combination with cladribine, idarubicin, and cytarabine; any previous treatment	All AML, MDS; age ≥ 18 (R/R cohort)
NCT04209725	II	Combination with liposomal cytarabine and anthracycline (CPX-351; Vyxeos); R/R after any prior treatment, first-line treatment must have been standard induction chemotherapy	*FLT3*-ITD AML; age 18−75
NCT04128748	I/II	Combination with liposomal cytarabine and anthracycline (CPX-351; Vyxeos); R/R to first, second, third, or fourth line therapy	All AML, MDS; age ≥ 18
NCT04112589	I/II	Combination with FLAG-Ida (fludarabine, cytarabine, idarubicin, GCSF); R/R to frontline standard induction chemotherapy	All AML; age 18−70
**Crenolanib**			
NCT03250338	III	Crenolanib vs Placebo plus salvage chemotherapy; R/R after first or second-line treatment, including HCT	*FLT3-*ITD and -D835; age 18−75

### Stratification of FLT3 Inhibitors

First generation FLT3 inhibitors include sorafenib, midostaurin, lestaurtinib, sunitinib, and tandutinib. These multi-kinase inhibitors target not only FLT3 but other kinases as well, including PKC, SYK, FLK-1, AKT, PKA, KIT, FGR, SRC, PDGFRα/β, and VEGFR 1/2 (midostaurin) and RAF, VEGFR 1/2/3, PDGFRβ, KIT, and RET (sorafenib). The antileukemic effects of these non-specific inhibitors likely derive not only from FLT3 inhibition, but from inhibition of targets in these parallel pathways as well. Similarly, due to their multiple off-target effects, first generation FLT3 inhibitor may also be associated with increased toxicities. In contrast, second generation FLT3 inhibitors are more potent, FLT3-specific and thus have fewer off-target effects at clinically relevant doses. Second generation FLT3 inhibitors include gilteritinib, quizartinib, and crenolanib.

FLT3 inhibitors can also be subdivided based upon how they interact with the intracellular kinase domain (KD) of the FLT3 receptor. In normal physiology, FLT3 ligand binds to the extracellular domain, causing the FLT3 receptor to dimerize, assume an enzymatically active conformation and subsequently activate downstream signaling. Type I FLT3 inhibitors, which include midostaurin, gilteritinib, lestaurtinib, and crenolanib, bind the receptor in the active conformation, thus inhibiting both *FLT3-*TKD and *FLT3-*ITD mutated receptors. Type II inhibitors, including quizartinib and sorafenib, bind to a region adjacent to the ATP-binding pocket and only inhibit the receptor in the inactive conformation. Type II inhibitors are inactive against most *FLT3*-TKD mutations as these mutations bias the active kinase conformation of FLT3. This distinction is key in understanding mechanisms of FLT3 inhibitor resistance, as discussed later in this review.

## First-Generation FLT3 Inhibitors

### Midostaurin

Midostaurin was one of the first *FLT3* inhibitors to be studied in AML. Early studies of single-agent midostaurin in R/R AML demonstrated the drug was well-tolerated, but had limited efficacy (19, 31).

Results of midostaurin in combination therapy have been more promising. In the phase III RATIFY trial, midostaurin was evaluated in combination with standard induction and consolidation chemotherapy in adults <60 years with *FLT3*-mutated AML. This combination demonstrated significant improvement in the primary endpoint of overall survival (OS), with a median OS of 74.7 months in patients receiving midostaurin plus chemotherapy *vs* 25.6 months in patients receiving placebo plus chemotherapy (p = 0.009) (20). In 2017, over two decades since *FLT3* mutations were first described in AML, midostaurin gained US Food and Drug Administration (FDA) approval, becoming first agent to achieve FDA approval for AML since the year 2000.

While the results of RATIFY were promising, there are some important caveats to consider. In RATIFY, 23% of the study population had a *FLT3-*TKD mutation, significantly larger than that seen in the general population, and perhaps biasing the results toward this less-aggressive disease subtype. In addition, while patients in RATIFY were younger (median age 48), FDA approval was extended to all age groups (20). The phase II AMLSG 16-10 trial is currently evaluating midostaurin in combination with induction and consolidation chemotherapy in adults up to age 70 (NCT01477606), with initial results suggesting that older age does not significantly impact outcomes, despite the majority of patients requiring midostaurin dose reduction (32).

### Sorafenib

Unlike RATIFY, trials of sorafenib in combination with chemotherapy have not been conducted in only *FLT3*-mutant patients and can thus be more challenging to interpret. Early phase I/II results of sorafenib in combination with induction chemotherapy demonstrated promising results, with a 75% complete response (CR) rate in all patients and a 93% CR rate in the subset with *FLT3-*ITD mutant AML (22). These findings, however, were not replicated in follow-up randomized studies. In 2013, sorafenib plus chemotherapy was evaluated in older adults (61–80 years), but did not show benefit in either the primary endpoint of EFS or in OS, including in *FLT3*-ITD subgroup analysis (median EFS 7 *vs* 5 months, p = 0.12). Furthermore, sorafenib demonstrated marked toxicity, presumably due to off-target effects (33).

In 2015, sorafenib plus chemotherapy was evaluated in adults <60 years in the randomized phase II SORAML trial. While sorafenib demonstrated improvement in the primary endpoint of EFS compared to placebo in all patients regardless of *FLT3* status (median EFS 21 *vs* 9 months, p = 0.013) (23), there was ultimately no difference in OS (5y OS 61 *vs* 52%, p = 0.23) (34).

Sorafenib may have efficacy as a single agent. Early phase I and retrospective studies of sorafenib monotherapy in R/R AML irrespective of *FLT3* status demonstrated acceptable toxicity profiles but mixed CR rates, ranging from 10 to 48% (35, 36). In studies of *FLT3*-ITD R/R AML, response rates of sorafenib monotherapy were 23–92% (37–39) with some post-transplant patients achieving sustained remission for multiple years (39, 40). Sorafenib does not have regulatory approval for AML but can be used off-label in the US as it is approved for hepatocellular, renal cell, and thyroid cancer.

### Sunitinib, Lestaurtinib, Tandutinib

Other first-generation FLT3 inhibitors have demonstrated limited antileukemic effect as monotherapy and mixed results when combined with chemotherapy. As single-agent therapy for patients with R/R disease, sunitinib (41), lestaurtinib (42), and tandutinib (43) have all demonstrated limited and short-lived responses. In combination with chemotherapy, a phase I/II study of sunitinib plus frontline chemotherapy in adults >60 demonstrated a 59% CR rate; however, multiple patients experienced dose-limiting toxicities (44). To contrast, a randomized phase III trial of lestaurtinib plus frontline induction and consolidation chemotherapy in patients with *FLT3*-mutated AML demonstrated no difference in primary endpoints of OS (5-year OS 46% lestaurtinib *vs* 45% control, p = 0.3) or RFS (5-year RFS 40 *vs* 36%, p = 0.30) (45). Similarly, a randomized phase III trial of chemotherapy with or without lestaurtinib in patients with *FLT3-*mutated AML in first relapse demonstrated no difference in the primary endpoint of CR rates (26 *vs* 21%, p = 0.35) (42). Currently, none of these agents are approved for AML and development in AML has been abandoned.

## Second-Generation FLT3 Inhibitors

Unlike midostaurin and sorafenib, second-generation *FLT3* inhibitors are more specific to the FLT3 receptor, exhibit greater potency, and have thus been far more efficacious as single-agent therapy. Both gilteritinib and crenolanib are type I inhibitors, active against both the active and inactive conformation of the FLT3 receptor, while quizartinib is a type II inhibitor, active only against the inactive form.

### Quizartinib

Quizartinib has demonstrated improved potency and selectivity against FLT3-ITD in preclinical cellular assays, and targets KIT and PDGFR as well as FLT3 (46). Early phase II studies of quizartinib monotherapy in R/R disease were highly promising, with CR rates of 46–56% and median OS of 25 weeks in *FLT3*-ITD mutated AML (25, 47). Quizartinib also demonstrated an acceptable safety profile aside from QTc prolongation, leading to an additional phase IIb study which explored dose reduction to 60 and 90 mg daily *vs* up to 200 mg daily (47).

Quizartinib was subsequently evaluated in the phase III randomized QuANTUM-R trial, which randomized patients with R/R *FLT3*-ITD AML to single-agent quizartinib *vs* salvage chemotherapy (26). Quizartinib was associated with a significantly longer primary endpoint of overall survival (6.2 *vs* 4.7 months, p = 0.02), and a greater proportion of patients were able to proceed to hematopoietic cell transplantation (HCT; 32 *vs* 11%). Total treatment-associated toxicities were similar between the two arms, although 2% of patients receiving quizartinib experienced Grade 3 QTc prolongation. Based on these results, the authors of QuANTUM-R concluded that quizartinib monotherapy should be considered a standard of care option in R/R *FLT3*-ITD mutated AML (26).

Despite these positive results, in 2019, both the FDA and the European Medicines Agency (EMA) rejected approval for quizartinib, although regulatory approval was granted in Japan. Although the FDA Oncologic Drug Advisory Committee (ODAC) raised concerns that up to four deaths in the quizartinib arm were attributed to cardiotoxicity, the decision to decline was ultimately due to concerns regarding trial design (48). These concerns included (1) an imbalance in patients who were randomized but not treated, with 23% of patients randomized to chemotherapy not receiving treatment *vs* 11% of patients randomized to quizartinib and (2) in stratified analysis, a significant survival benefit only when quizartinib was compared against low-intensity therapy (low-dose cytarabine) and not against high-intensity therapy (MEC or FLAG-Ida). The ODAC concluded that, while a modest survival benefit of 6 weeks was still meaningful, especially if more patients were bridged to HCT, ultimately additional data would be needed for quizartinib to be approved in this setting (48). The phase II Q-HAM trial, which has not yet begun recruiting, will evaluate quizartinib in combination with salvage chemotherapy in R/R *FLT3*-ITD AML (NCT03989713).

In newly diagnosed AML, quizartinib is currently being evaluated in the randomized phase III QuANTUM-First trial, which will compare quizartinib *vs* placebo in combination with induction and consolidation chemotherapy (NCT02668653). Additional phase I/II studies evaluating quizartinib in combination with frontline cytotoxic chemotherapy are ongoing as well (NCT03723681, NCT03135054, NCT04047641).

### Gilteritinib

Gilteritinib is a selective and potent type I FLT3 inhibitor which also has activity against AXL, ALT, and ALK (49). Gilteritinib is a particularly promising agent due to its ability to target KD mutations, including the D835 residue, the development of which is a key mechanism of both quizartinib (50) and sorafenib (51) resistance. In the phase I/II CHRYSALIS trial, gilteritinib demonstrated promising results as monotherapy in R/R FLT3-mutant AML with an overall response rate (ORR) of 40% and a median OS of 25 weeks (52). Notably, patients with FLT3-D835 mutations also responded to gilteritinib, albeit at lower rates than patients with FLT3-ITD mutations, with an ORR of 55% in *FLT-*ITD-mutated patients, 17% in *FLT3*-D835-mutated patients, and 62% in patients with both *FLT3*-ITD and *FLT-*D835 mutations (52).

Following these results, the randomized phase III ADMIRAL trial compared single-agent gilteritinib *vs* salvage chemotherapy in R/R *FLT3-*mutated AML (27) with co-primary endpoints of CR rate and OS. Compared to standard salvage chemotherapy, gilteritinib demonstrated significantly greater CR rate (34 *vs* 15%, p = 0.0001) and improvement in OS (9.3 *vs* 5.6 months, p < 0.001). While prior use of midostaurin or sorafenib was allowed, as the trial enrolled prior to midostaurin’s approval and widespread use, the majority (88%) of patients had not received a prior FLT3 inhibitor, limiting the ability of this trial to evaluate the ability of gilteritinib to overcome midostaurin resistance; however, results were similar in patients with *FLT3-*ITD and *FLT3-*TKD mutated disease (27). Based on a pre-planned interim analysis, in 2018, the FDA approved gilteritinib as single-agent therapy for R/R *FLT3-*mutated AML.

It is unknown whether gilteritinib is similarly beneficial in newly diagnosed AML. Gilteritinib is currently being studied in a phase I study in combination with induction and consolidation chemotherapy in newly diagnosed AML (NCT02236013); interim results indicate this approach is safe and feasible (53). The phase III HOVON 156 trial, which is actively accruing, will compare gilteritinib *vs* midostaurin in combination with chemotherapy followed by FLT3 inhibitor maintenance (NCT04027309).

### Crenolanib

Crenolanib is a potent type I inhibitor with activity against PDGFRβ, *FLT3-*ITD, and *FLT3-*TKD mutations, including at D835 (54). Two smaller phase II studies have demonstrated efficacy of single-agent crenolanib in R/R *FLT3-*mutated AML, with a CR rate of 23−39% in patients naïve to FLT3 inhibitors, and 5% patients with prior FLT3 exposure (29, 30).

Crenolanib also demonstrates promising results in combination with chemotherapy. A phase II trial of crenolanib plus chemotherapy in newly diagnosed *FLT3-*mutated AML demonstrated a high CR rate of 85%; notably, 70% of patients remained alive and disease free with a median follow-up of 29.3 months (55). Crenolanib plus chemotherapy is also efficacious in older adults. In a phase II trial of adults 61–75 years with newly diagnosed *FLT3-*mutated AML, crenolanib plus chemotherapy demonstrated a CR rate of 86% and was relatively well-tolerated, although four of 14 patients did require dose-reductions due to toxicity (56). A phase III trial of crenolanib *vs* midostaurin plus chemotherapy in newly diagnosed *FLT3-*mutated AML is currently recruiting (NCT03258931).

## Resistance to FLT3 Inhibitors

**Figure 1:**
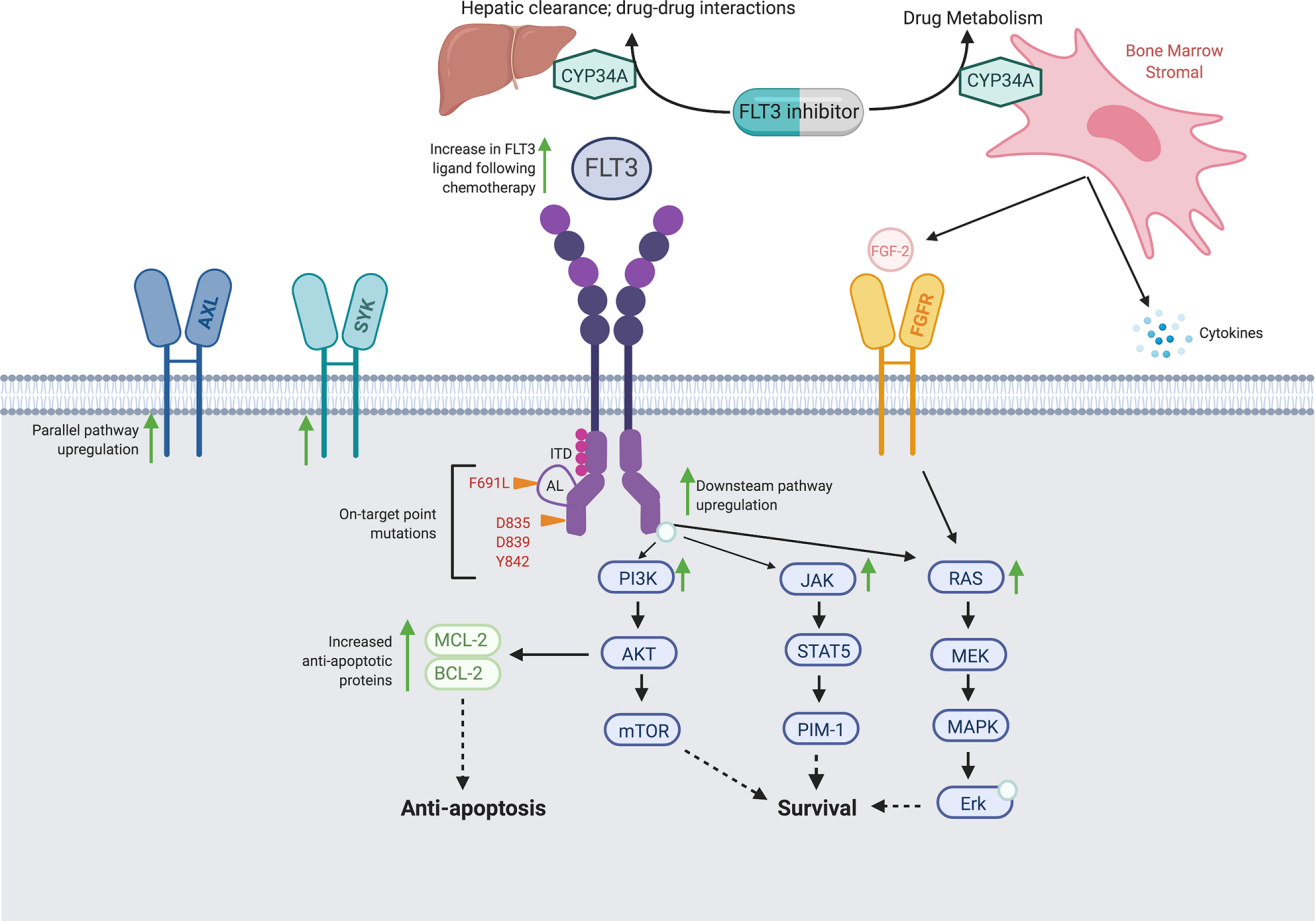
Proposed Intrinsic and Extrinsic Mechanisms of FLT3 Inhibitor Resistance. Schematic of FLT3 inhibitor resistance mechanisms, including on-target secondary *FLT3* mutations, off-target mutations in parallel and/or downstream signaling pathways, and extrinsic alterations in drug metabolism and the bone marrow microenvironment.

Despite the relative success of established FLT3 inhibitors, responses are frequently short-lived and therapeutic resistance poses an ongoing challenge. Mechanisms of FLT3 inhibitor resistance differ based on drug type, but broadly can be subdivided into cell intrinsic and extrinsic mechanisms. Intrinsic resistance can be further sub-divided into on-target secondary mutations within *FLT3* and off-target mutations in downstream or parallel signaling pathways. These mutations may develop *de novo* or *via* expansion of pre-existing subclones (57). Extrinsic resistance can occur through altered expression or metabolism of the FLT3 ligand or changes in the interactions between the leukemic blast and the bone marrow microenvironment. **Figure 1** illustrates common resistance pathways and mechanisms.

### On-Target Secondary Mutations

One common mechanism of FLT3 inhibitor resistance is development of a secondary *FLT3* mutation, most commonly in the KD (58). These mutations commonly occur at gatekeep F691 and activation loop (AL) D835 residues, but can involve other KD residues I836, D839, and Y842, among others (59). This mechanism is most relevant for type II FLT3 inhibitors, which interact weakly with the active receptor formation caused by some KD mutations. Type I inhibitors gilteritinib and crenolanib are both active against D835 mutations (60, 61).

To contrast, the gatekeeper F691L mutation confers resistance not only to quizartinib and sorafenib, but also to gilteritinib and crenolanib as well (51, 60–62). In practice, the impact of F691L mutations on type I inhibitor resistance may be relatively minor. In studies of both single-agent gilteritinib (62, 63) and single-agent crenolanib (64) resistance in patients with R/R *FLT3-*mutated AML, only 12% of patients receiving gilteritinib, and 11% of patients receiving crenolanib developed F691L mutations at the time of resistance.

In most cases, on-target mutations are not detected prior to FLT3 inhibitor treatment (65). In a recent study of 11 patients treated with quizartinib monotherapy, single-cell sequencing revealed 7/11 patients developed KD mutations at relapse and no patients had these mutations prior to FLT3 inhibition, suggesting TK mutations typically evolve *de novo* (66) or exist at levels undetectable by current sequencing methods.

### Off-Target Resistance in Parallel or Downstream Pathways

KD mutations only partly explain FLT3 inhibitor resistance, and expansion or emergence of non-*FLT3* mutant clones is a key resistance mechanism. In patients treated with gilteritinib and crenolanib, sequencing of paired patient samples pre- and post-therapy demonstrated a wide variety of mutations at resistance, including genes involved in the RAS pathways (*NRAS, KRAS, PTPN11)*, *ASXL1, TP53, TET2*, and *IDH1/2*. These mutations occurred not only in cells expressing the *FLT3-*mutant allele, but in *FLT3-*WT cells as well (63, 64). These off-target mutations are not limited to type I inhibitors. In a study using single-cell sequencing to analyze 11 patients treated with quizartinib, 3/11 demonstrated off-target mutations at relapse, all of which were present in small clonal populations prior to FLT3 inhibitor therapy (66).

Of these off-target pathways, activation of the downstream Ras and associated PI3K/Akt/mTOR and MAPK/Erk pathways are particularly common in clinical gilteritinib and crenolanib resistance (62–64), and *in vitro* studies have demonstrated mutations in these pathways can confer resistance to FLT3 inhibitors in FLT3-mutant cell lines (67, 68). Development of the *BCR-ABL1* fusion gene has been describe in patients with gilteritinib resistance (63, 69).

Upregulation of parallel AXL tyrosine kinase signaling is another mechanism of FLT3 inhibitor resistance. In one study, blasts from a patient with *FLT3-*ITD AML were exposed to both midostaurin and quizartinib and were found to have increased AXL phosphorylation upon development of FLT3 inhibitor resistance. This resistance could be overcome with use of the AXL inhibitor TP-0903 (70, 71) and a phase I trial of TP-0903 with or without azacitidine in *FLT3-*mutated AML has recently opened (NCT04518345).

Together, these studies suggest off-target resistance mechanisms are common to all FLT3 inhibitors and frequently arise *via* selection of pre-existing sub-clones harboring survival advantages under the selective pressure of FLT3 inhibition.

### Extrinsic Mechanisms of Resistance

The majority of leukemic blasts, regardless of mutational status, express the FLT3 receptor and proliferate in response to FLT3 ligand. Increased levels of FLT3 ligand in the bone marrow microenvironment have been demonstrated during induction and consolidation chemotherapy, and can lead to increased signaling *via* the native FLT3 receptor, even in the presence of FLT3 inhibitors (72, 73). FLT3 inhibitors may also have decreased efficacy due to decreased drug availability, either due to enhanced CYP34A expression on BM stromal cells (74) or iatrogenic drug-drug interactions (75).

The bone marrow microenvironment can also directly contribute to FLT3 inhibitor resistance. In addition to directly secreting FLT3 ligand, bone marrow stromal cells can also upregulate Ras/MAPK signaling independent of the FLT3 receptor *via* secretion of FGF2 (76). In one study of patients treated with quizartinib, stromal cell expression of FGF2 conferred FLT3 inhibitor resistance, and could be overcame by FGFR inhibition (76, 77). Multiple dual FLT3/FGFR inhibitors are in pre-clinical development (78, 79) with MAX-40279 currently in phase I clinical trials (NCT03412292, NCT04187495). In addition, ponatinib, a multikinase inhibitor approved in CML, has activity against both FLT3 and FGFR (80).

## Novel FLT3 Inhibitor Combinations

**Figure 2:**
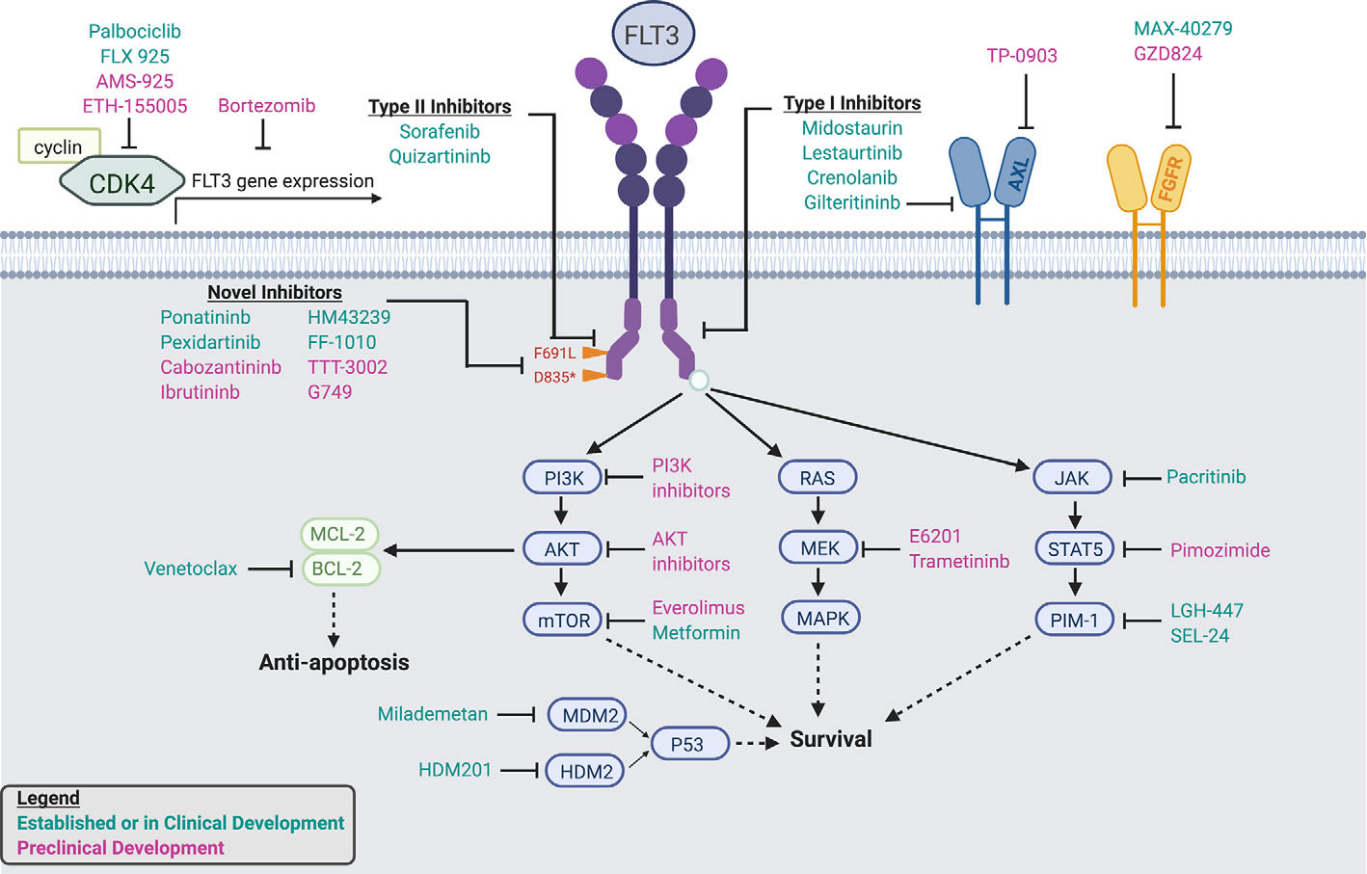
Established and In-Development FLT3 inhibitors, dual inhibitors, and combination agents. Schematic detailing mechanisms of action of established and in-development FLT3 inhibitors, dual and multikinase inhibitors, and combination agents.

One strategy to overcome resistance and provide durable remissions is to use FLT3 inhibitors in novel combinations with other antileukemic agents (**Tables 4**, **5**; **Figure 2**).

**Table 4:** Select Trials of Established FLT3 Inhibitors in Combination Therapy.

Combination Agent	FLT3 inhibitor	NCT/Trial Identified	Phase	Treatment Setting	Patient Population
**Hypomethylating Agents**					
Azacitidine	Midostaurin	NCT01093573	I/II	Newly diagnosed	All AML; age ≥ 18 and unfit for chemotherapy
Decitabine	Midostaurin	NCT04097470; HO-155	II	Midostaurin plus decitabine vs decitabine alone, newly diagnosed	All AML, age 18–100 and unfit for chemotherapy
Azacitidine	Sorafenib	NCT02196857	II	Newly diagnosed	*FLT3-*ITD, TKD AML, MDS; age ≥ 60 or 18−60 and unfit for chemotherapy
Azacitidine	Gilteritinib	NCT02752035; LACEWING	II/III	Gilteritinib monotherapy vs azacitidine monotherapy vs gilteritinib plus azacitidine; newly diagnosed AML	*FLT3-*ITD, TKD; age ≥ 65 or 18–65 and unfit for chemotherapy
Azacitidine or Low-Dose AraC	Quizartinib	NCT01892371	I/II	Quizartinib plus azacitidine or cytarabine; Newly diagnosed or R/R after first or second-line treatment, including HCT	All AML, MDS, CMML; age ≥ 60 (all settings) or age ≥ 18 (R/R only)
**Venetoclax +/- HMA**					
Venetoclax	Gilteritinib	NCT03625505	I	R/R to at least one prior therapy	All AML; age ≥ 18
Venetoclax + Azacitidine	Gilteritinib	NCT04140487	I/II	Newly diagnosed or R/R	*FLT3-*ITD or D835 AML; age ≥ 18
Venetoclax + Decitabine	Quizartinib	NCT03661307	I/II	Newly diagnosed; R/R and not eligible for salvage chemotherapy or HCT	*FLT3-*ITD or *FLT3-*ITD/TKD co-mutations; age ≥ 60 or age ≥ 18 and unfit for chemotherapy
Venetoclax	Quizartinib	NCT03735875	Ib/II	R/R up to 4 prior therapies	*FLT3-*ITD; age ≥ 18
**Proteosome Inhibitors**					
Bortezomib	Sorafenib	NCT01371981	III	Bortezomib plus sorafenib plus chemotherapy vs sorafenib plus chemotherapy; Newly diagnosed	*FLT3-*ITD; age < 29
Bortezomib, then decitabine	Sorafenib	NCT01861314	I	Newly diagnosed, R/R to at least one prior therapy	All AML; age ≥ 60 or ≥ 18 and unfit for chemotherapy
Bortezomib, Vorinostat	Sorafenib	NCT01534260	I/II	R/R to at least one prior therapy	*FLT3-*mutated or poor-risk cytogenetics; age ≥ 18
**Targeted Agents**					
Pim kinase inhibitor (LGH447)	Midostaurin	NCT02078609	I	R/R after first or second-line treatment	All AML, MDS; age ≥ 18
mTOR inhibitor (Everolimus)	Midostaurin	NCT00819546	I	R/R to at least one prior therapy	All AML, MDS; age ≥ 18
HDM2 inhibitor (HDM201)	Midostaurin	NCT04496999	I	R/R to at least one prior therapy	*FLT3-*ITD or *FLT3-*TKD and *TP53*-WT; age ≥ 18
CDK 4/6 inhibitor (Palbociclib)	Sorafenib	NCT03132454	I	Palbociclib in combination with sorafenib vs decitabine vs dexamethasone, newly diagnosed	All AML, ALL; age ≥ 15
MDM2 inhibitor (Milademetan)	Quizartinib	NCT03552029	I	Newly diagnosed and ineligible for intensive therapy; R/R to at least one prior therapy	*FLT3-*ITD; age ≥ 18
**Immunotherapy**					
Atezolizumab	Gilteritinib	NCT03730012	I/II	R/R to at least one prior therapy	*FLT3-*mutated AML; age ≥ 18

**Table 5:** Select Trials of Novel FLT3-Directed Therapies.

Combination Agent	NCT	Phase	Treatment Setting	Study Population
**Multikinase Inhibitors**				
Ponatinib	NCT02428543	I/II	AML in first CR, following induction and consolidation with standard cytotoxic chemotherapy	*FLT3-*ITD with AR > 10%; age 18−70
Ponatinib	NCT03690115; PONALLO	II	AML in CR, following allo-HCT	*FLT3-*ITD; age 18 - 70
**Novel Dual Agents**				
SEL24/MEN1703: Dual FLT3/Pim kinase inhibitor	NCT03008187	I	R/R, no standard treatment options available	All AML; age ≥ 18
MAX-040279: Dual FGFR/FLT3 inhibitor	NCT03412292	I	R/R, no standard treatment options available	All AML; age ≥ 18
MAX-040279: Dual FGFR/FLT3 inhibitor	NCT04187495	I	R/R, no standard treatment options available	All AML; age ≥ 18
CG-806: Dual BTK/FLT3 inhibitor	NCT04477291	Ia/b	R/R to at least one prior therapy	All AML; age ≥ 18
**Novel FLT3 Inhibitors**				
FF-10101	NCT03194685	I/IIa	R/R to at least one prior therapy	All AML; age ≥ 18
HM43239	NCT03850574	I/II	R/R to at least one prior therapy	All AML; age ≥ 18
**Biologic Agents**				
FLYNSYN: Anti-FLT3 IgG1 Antibody	NCT02789254; FLYSYN-101	I/II	AML in and hematologic CR but MRD+ after chemotherapy but not HCT	All AML, but leukemic cells must express FLT3 by flow cytometry; age ≥ 18
ASP1235 (AGS62P1): anti-FL3 antibody-drug-conjugate	NCT02864290	I	R/R to first, second, or third therapy	All AML; age ≥ 18 and not candidate for salvage chemotherapy
AMG 553: FLT3 CART	NCT03904069	I	R/R, no standard treatment options available	All AML, but leukemic cells must express FLT3 by flow cytometry; age ≥ 12

### Hypomethylating Agents

Aside from conventional cytotoxic chemotherapy, the most well-studied FLT3 inhibitor combination is with the hypomethylating agents (HMA) azacitidine or decitabine. This combination is particularly attractive, both due to the synergistic cytotoxicity in preclinical studies as well as the established tolerability and durable responses of HMAs in older adults (81, 82).

Multiple phase I/II trials have demonstrated the combination of midostaurin and HMA to be feasible in adults with *FLT*-mutated AML who are unfit for traditional chemotherapy in the frontline setting (83) and in R/R disease (82, 84). Additional trials of midostaurin plus HMA for older/unfit adults have opened, but have terminated due to inability to accrue (NCT01846624. NCT02634827). There is an ongoing trial of midostaurin plus azacitidine for newly diagnosed AML regardless of *FLT3* mutational status (NCT01093573), with primary endpoints of tolerability and ORR.

Sorafenib plus HMA have also been shown to be safe and efficacious in single-arm and retrospective studies in both the R/R (85, 86) and frontline settings (87). In the frontline setting, sorafenib plus HMA demonstrated an ORR of 78%, the study’s primary endpoint, and a and median response duration of 14.5 (87). Similarly, a phase I/II study of sorfenib plus azacitidine demonstrated an ORR of 46%, the secondary endpoint for the trial’s phase II portion, and median response duration of 2.3 months (85). Notably, FLT3 ligand levels remained low with this combination, which is intriguing as increase in ligand expression has been suggested as a possible mechanism of FLT3 inhibitor resistance (72, 85).

Of the second generation FLT3 inhibitors, an interim analysis of a phase I/II trial of quizartinib plus azacitidine in unfit patients with newly diagnosed or in R/R *FLT3-*ITD AML demonstrated an ORR of 75%, the secondary endpoint for the trial’s phase II portion, including in four/five patients with prior FLT3 inhibitor exposure (88). A phase II study of gilteritinib plus azacitidine in unfit patients with newly diagnosed *FLT3-*ITD AML with primary endpoint of OS is currently accruing (NCT02752035) with interim results of secondary endpoints demonstrating CR and ORR rates of 67 and 80%, respectively (89).

### Venetoclax

Venetoclax, an inhibitor of the anti-apoptotic protein Bcl-2, is particularly intriguing in combination with FLT3 inhibitors. Preclinical studies have shown *FLT3*-ITD mutated blasts have higher Bcl-2 expression compared to *FLT3*-WT blasts (90) and upregulation of antiapoptotic proteins is a mechanism of FLT3 inhibitor resistance (91). In preclinical mouse models, venetoclax has demonstrated synergistic antileukemic activity with midostaurin (92), gilteritinib (92), and quizartinib (93). In addition, *FLT3*-ITD mutations were frequently observed at progression in trials of venetoclax monotherapy in AML (94, 95).

Gilteritinib is currently being studied in combination with venetoclax in R/R AML (NCT03625505). Quizartinib is being evaluated in combination with venetoclax and decitabine in poor-risk newly diagnosed or R/R *FLT3-* mutated AML (NCT03661307) and in combination with venetoclax alone in R/R *FLT3*-ITD AML (NCT03735875).

### Proteosome Inhibitors

Proteosome inhibitors, including bortezomib, have demonstrated cytotoxicity toward *FLT3-*ITD mutant cells in preclinical studies (96) and a phase I study of midostaurin in combination with bortezomib and chemotherapy in R/R AML demonstrated activity, albeit with marked toxicity (97). Sorafenib plus bortezomib was also studied in a combination with vorinostat, a histone deacetylase inhibitor, in a phase I/II trial of R/R AML and demonstrated a modest ORR of 28% in all patients and 60% in patients with *FLT3-*ITD AML (98). Ongoing studies of this combination include a phase III trial of sorafenib plus bortezomib in younger adults (up to age 29) with newly diagnosed *FLT3-*ITD AML (NCT01371981) and sorafenib plus bortezomib followed by decitabine in newly diagnosed or R/R AML regardless of *FLT3* status (NCT01861314).

### Additional Targeted Agents

Multiple agents targeting signaling pathways downstream of FLT3 signaling, including those frequently implicated in FLT3 inhibitor resistance, have been studied in combination therapies.

JAK/STAT5/Pim-1 is a key signaling pathway parallel and downstream of FLT3. **Pim kinase inhibitors** inhibit Pim-1, a kinase which promotes FLT3 signaling *via* positive feedback (99, 100). In preclinical models, Pim kinase and FLT3 inhibitors demonstrate synergistic cytotoxicity (100, 101). The Pim kinase inhibitor LGH447 is being studied in combination with midostaurin in a phase I trial (NCT02078609), as is the novel dual Pim kinase/FLT3 inhibitor SEL24 (NCT03008187). Other agents, including the dual JAK/FLT3 inhibitor pacritinib, have demonstrated efficacy in phase I trials as well (102).

**mTOR inhibitors**, such as everolimus, target the PI3K/AKT/mTOR pathways, which is similarly downstream of FLT3 signaling. Concomitant inhibition of both mTOR and FLT3 demonstrate synergistic cytotoxicity (103) and mTOR is upregulated in AML blasts resistant to FLT3 inhibitors (68). A phase I study of midostaurin plus everolimus is active, but not currently recruiting (NCT00819546). In addition, metformin, a drug long approved in diabetes, can also down-regulate the P31K/Akt/mTOR pathways, and has shown to act synergistically with sorafenib in FLT3-mutated cell lines (104).

Cyclin-dependent kinase 6 (CKD6) is a key regulator of cell cycle progression, part of a transcriptional complex that promotes leukemogenesis, and is found at the promoter of both *FLT3* and *PIM1* genes (105, 106). A phase I study of the **CDK4/6 inhibitor** palbociclib, which is approved in breast cancer, in combination with sorafenib is actively recruiting (NCT03132454). In addition, multiple novel dual FLT3/CDK4 inhibitors are in active preclinical development, including AMS-925 (107) (Keegan), ETH-155008 (108), and FLX925 (109), which recently completed a phase I dose-escalation trial (NCT02335814).

Finally, the tumor suppressor p53 is increasingly recognized as a mechanism of FLT3 inhibitor resistance, particularly to crenolanib (64). Milademetan, an inhibitor of the p53-regulatory protein **MDM2**, has demonstrated synergistic anti-leukemic activity with quizartinib in FLT3-mutant cell lines (110) and a phase I trial is actively recruiting (NCT03552029). Similarly, HDM-201, an inhibitor of the p53-regulatory protein **HDM2**, is actively investigated in combination with midostaurin (NCT04496999).

#### Immunotherapy Combinations

Compared to lymphoid and many solid malignancies, AML has thus far demonstrated limited response to immunotherapies. Exploratory studies have indicated that elevated programmed cell death 1 (PD-1) and PD-1 ligand (PD-L1) are associated with inferior OS in AML, including in patients with *FLT3-*mutated disease (111). A phase I/II trial of gilteritinib in combination with the checkpoint inhibitor atezolizumab in R/R FLT-mutated AML is ongoing (NCT03730012).

## Novel FLT3 Inhibitors in Development

In addition to novel FLT3 inhibitor combinations, multiple novel FLT3 inhibitors are in active preclinical and clinical development (**Table 5**; **Figure 2**). These include multikinase inhibitors approved for non-AML malignancies as well as specific, next-generation agents. In addition to overcoming pre-established mechanisms of resistance, including F691 mutations, these novel agents also offer alternative and potentially more desirable toxicity profiles.

### Multikinase Inhibitors

**Ponatinib** is approved to target BCR-ABL in chronic myelogenous leukemia (CML), but is also a type II FLT3 inhibitor with activity against F691L (112). Ponatinib demonstrated modest efficacy in a phase I trial of heavily-pretreated R/R AML patients (113), and is being actively investigated in combination with chemotherapy (NCT02428543).

**Cabozantinib** is a multikinase inhibitor currently approved for medullary thyroid and renal cell carcinomas. Cabozantinib is selectively cytotoxic to *FLT3-*ITD mutant cells in culture (114) and a phase I trial demonstrated sustained inhibition of *FLT3-ITD* and *-F691* mutant cells, although no treated patients had a formal disease response (115).

The multikinase inhibitor **pexidartinib** (PLX3397) has been studied in multiple solid tumors and also demonstrates activity against FLT3, including F691L (50). Recently, a phase I/II trial of single-agent pexidartinib in R/R *FLT3-*ITD AML demonstrated an ORR of 21% and CR rate of 11%; furthermore, several patients were successfully bridged to transplant (116).

Finally, the BTK inhibitor **ibrutinib**, currently approved for lymphoid malignancies, also demonstrates type II FLT3 inhibitory effects (117). A phase I trial of CG-806, a dual BTK/FLT3 inhibitor, recently opened for patients with R/R AML (NCT04477291).

### Novel FLT3 Inhibitors

One of the most promising novel agents is **FF-10101**, the first covalently-binding FLT3 inhibitor. In pre-clinical studies, FF-10101 demonstrated potent activity against quizartinib-resistant AL and gatekeeper F691 mutations (118). Other agents in development include TTT-3002, G-749, MZH-29, and HM43239, all highly selective FLT3 inhibitors with activity against D835 and F691 residues (119–122). In preclinical studies, these agents have demonstrated activity against AML blasts resistant to sorafenib and quizartinib (119) or midostaurin and quizartinib (120), and may represent options for refractory disease. Phase I/II trials of both FF-10101 and HM43239 are ongoing (NCT03194685, NCT03850574).

### Biologic Agents

To date, pharmacologic targeting of FLT3-mutant AML has primarily focused on signaling inhibition *via* small molecules; however, multiple immunotherapeutic approaches are in development.

Ongoing trials are investigating targeting FLT3 through an IgG1 antibody (FLYSYN; NCT02789254), with promising preliminary efficacy data (123), as well as *via* an anti-FLT3 antibody drug conjugate (124) (NCT02864290). In addition, an anti-FLT3/anti-CD3 bi-specific antibody has shown promise in preclinical models as well (125). Finally, FLT3 may represent a potential target for chimeric antigen receptor T cell (CART) therapy (126), including in combination with established FLT3 inhibitors (127). A phase I trial of a FLT3-directed CART has recently opened (NCT03904069).

## Ongoing Questions and Controversies

Despite the considerable advances in treating *FLT3-*mutant AML, many outstanding questions and controversies remain. We have summarized a few of the most intriguing and timely questions below.

### What is the Benefit of Maintenance Therapy in FLT3-Mutant AML?

Of all the questions regarding the clinical use of FLT3 inhibitors, one of the most pressing is the role of maintenance therapy. Use of FLT3 inhibitor maintenance, either during remission for patients who do not undergo HCT or during post-HCT remission, is currently not standard of care; however, there are data to suggest this may be a promising approach. **Table 6** describes ongoing clinical trials of maintenance therapy.

**Table 6:** Select Trials of FLT3 Inhibitors as Post-Chemotherapy or Post-HCT Maintenance Therapy.

FLT3 inhibitor	NCT/Trial Identifier	Phase	Treatment Setting	Patient Population
**Midostaurin**	NCT01477606/AMLSG 16−10	II	Combination with induction and consolidation chemotherapy, plus maintenance	*FLT3-*ITD; age <70
**Midostaurin/Gilteritinib**	NCT04027309/HOVON 156	III	Gilteritinib *vs* Midostaurin in combination with induction and consolidation chemotherapy, plus maintenance	*FLT3*-ITD or -TKD AML; age ≥18
**Midostaurin**	NCT03951961/MAURITUS	II	Midostaurin maintenance post-HCT; MRD + disease	*FLT3-*mutated; age ≥18
**Midostaurin/Crenolanib**	NCT03258931	III	Midostaurin vs Crenolanib post-HCT; MRD+ disease	*FLT3-*mutated; age 18−60
**Gilteritinib**	NCT02927262	III	Gilteritinib *vs* Placebo for up to 2 years following induction and consolidation chemotherapy	*FLT3-*ITD; age ≥18 with no plan for HCT
**Gilteritinib**	NCT02997202/BMT CTN 1506	III	Gilteritinib vs. Placebo following HCT; AML in CR1 or CRi1	*FLT3-*ITDi; age ≥ 18
**Quizartinib**	NCT02668653/QuANTUM-First	III	Quizartinib vs Placebo in combination with induction and consolidation chemotherapy, plus maintenance post-chemotherapy and post-HCT	*FLT3*-ITD AML; age 18−75
**Crenolanib**	NCT02400255	II	Crenolanib following HCT; AML in any CR by morphologic assessment	*FLT3-*ITD and -D835; age ≥18

### Post-Chemotherapy Maintenance

Perhaps most notably, in the experimental arm of the phase III RATIFY study, patients could receive up to one year of midostaurin maintenance following induction and consolidation chemotherapy plus midostaurin (20). In unplanned *post-hoc* analysis of the subset of patients who received either midostaurin or placebo maintenance, there was no benefit seen with midostaurin, although it was well-tolerated (128). Importantly, the median duration of midostaurin exposure was 3 months, as the majority (59%) of patients randomized to midostaurin received allogeneic HCT and thus received only two to three cycles of therapy, limiting the ability to draw conclusions from midostaurin maintenance data (20). Maintenance midostaurin after chemotherapy did not receive US FDA approval; however, regulatory approval for maintenance midostaurin was granted by the EMA.

Currently, both the single-arm AMLSG 16–10 trials, which will also evaluate midostaurin in combination with chemotherapy in older adults, and the phase III HOVON 156 AML trial, which will compare midostaurin *vs* gilteritinib plus chemotherapy in newly diagnosed AML, will each evaluate up to one year of midostaurin maintenance following chemotherapy (NCT01477606, NCT04027309). Of note, neither of these trials will directly compare maintenance midostaurin against placebo, which will make it difficult to isolate the true benefit of post-chemotherapy midostaurin maintenance.

Multiple ongoing trials are also evaluating second generation FLT3 inhibitors as post-chemotherapy maintenance. In the HOVON 156 AML trial, patients may receive up to one year of gilteritinib maintenance, although again, this study will not compare gilteritinib against a placebo control. A separate randomized phase III trial will compare gilteritinib vs placebo maintenance for up to two years following induction and consolidation therapy in patients who are not proceeding to HCT accrual (NCT02927262). Similarly, the QuANTUM-First trial will include up to three years of post-chemotherapy quizartinib maintenance and will be placebo-controlled.

### Post-HCT Maintenance

In the post-HCT setting, midostaurin was evaluated in a phase II trial as single-agent maintenance following midostaurin plus chemotherapy and subsequent HCT. Of the 56% of patients who ultimately received HCT and subsequent maintenance, midostaurin maintenance was associated with improved OS compared to a historical control group; however, given this comparison was relative to historic controls it must be interpreted with caution (129). Midostaurin maintenance was also associated with significant toxicities, particularly in older adults.

In the phase II RADIUS trial, patients were randomized to receive up to one year of maintenance midostaurin *vs* standard care following HCT. Although preliminary reports suggested that addition of midostaurin could reduce risk of relapse at 18 months post-HCT by 46%, 63% of patients receiving midostaurin required dose modifications and 25% discontinued midostaurin due to toxicity. RADIUS ultimately had inadequate enrollment to detect a statistically significant difference in 18-month relapse-free survival (RFS), the study’s primary endpoint (estimated 18-month RFS 89% in midostaurin arm *vs* 76% in standard-of-care arm, p = 0.27) (130).

Similarly, post-HCT sorafenib maintenance was shown to be tolerable and potentially efficacious in both retrospective and prospective phase I/II studies, although frequent dose adjustments were needed (131, 132). More recently, the phase II SORMAIN trial randomized patients with *FLT3*-ITD AML in remission following HCT to maintenance with two years of sorafenib *vs* placebo (133). The HR for RFS, the primary endpoint, demonstrated a significant benefit with sorafenib *vs* placebo (HR 0.39, 95% CI 0.18–0.85, p = 0.013); however, only 9/43 (21%) of patients receiving sorafenib had received a frontline FLT3 inhibitor, so it remains unclear whether the same benefit would be seen in patients who received a FLT3 inhibitor in the frontline setting. Similar to the RADIUS trial, study drug discontinuation was more common in the sorafenib arm (22 *vs* 5%), although this difference was not significant. Finally, and again similar to the RADIUS trail, SORMAIN did not reach is target accrual and was terminated prematurely.

Building on the results of RADIUS and SORMAIN, there the multiple ongoing trails evaluating post-HCT maintenance therapy. The phase II, single-arm MAURITUS trial will evaluate midostaurin maintenance following HCT in MRD-positive *FLT3*-mutated AML (NCT03951961) and a phase III trial with compare post-HCT midostaurin *vs* crenolanib maintenance (NCT03258931).

For the second generation FLT3 inhibitors, BMT CTN 1506, a randomized phase III trial of gilteritinib *vs* placebo for *FLT3*-ITD mutated AML following HCT is ongoing (NCT02997202). Importantly, although prior FLT3 inhibitor treatment is not an inclusion criterion, since enrollment occurred after the widespread use of midostaurin in the frontline setting, the majority of patients enrolled on BMT CTN 1506 will likely have received prior FLT3 inhibitor therapy, answering a key question raised by SORMAIN. In addition, quizartinib was shown to be well-tolerated as single-agent maintenance following HCT in a phase I study (134) and this strategy will be further explored in the QuANTUM-First trial. Finally, a non-randomized trial is evaluating the efficacy of crenolanib post-HCT in *FLT3-*mutated AML (NCT02400255).

### Maintenance Therapy: Where Do We Go From Here?

The role of FLT3 inhibitor maintenance, while promising, remains unknown, and placebo-controlled randomized trials are necessary to establish the efficacy of this approach. Multiple questions remain, including: How can we identify the patients for which FLT3 inhibitor maintenance is most beneficial? In SORMAIN, the strongest benefit from sorafenib maintenance was in patients with undetectable MRD pre-HCT and detectable MRD post-HCT (133). Notably, the ongoing BMT 1506 trial will include correlative MRD analysis to determine if the presence of MRD is predictive of benefit from FLT3 inhibitor maintenance. What is the optimal duration of maintenance therapy? In a correlative analysis of RADIUS, decreased levels of phosphorylated FLT3 were associated with improved OS (135), suggesting this may be one potential biomarker for determining maintenance duration. What type of FLT3 inhibitor is most efficacious as a maintenance agent, a multikinase, first-generation agent, or a more targeted, second generation one? In the post-chemotherapy setting, does maintenance serve as a bridge to transplant, or obviate the need? Finally, what is the effect of maintenance therapy on health-associated quality of life? Given that this patient population may not have active disease, studies specifically investigating health-associated quality of life are needed to fully inform the benefit of prolonged maintenance therapy.

#### What Is the Role of Transplant in FLT3-Mutant AML?

Historically, *FLT3*-mutated AML has been considered adverse risk disease, and patients with *FLT3-*ITD have been recommended to undergo HCT in the first CR (136, 137). More recently, the ELN has re-classified *FLT3-*mutated AML such that patients with *FLT3*-ITD^low^ (AR < 0.5), normal cytogenetics, and mutated *NPM1* are considered low risk, suggesting these patients may have good prognosis without HCT (138). This is not widely accepted, however, and retrospective data have demonstrated that ‘low risk’ *FLT3-*ITD AML, with *FLT3-*IT low AR and positive *NPM1* mutational status, still conveys poor prognosis, with a 5-year OS of 41.3%, and OS is improved by HCT (139). A similar retrospective study found that HCT in first CR improves OS in all *FLT3-*ITD AML, regardless of AR or *NPM1* status (140). Importantly, both these studies and the ELN guidelines were written prior to widespread FLT3 inhibitor use, and the role of HCT in *FLT3-*mutated AML today remains an open question.

In a retrospective analysis of the RATIFY trial, the beneficial effect of midostaurin was seen across all ELN subgroups; however, the benefit of HCT was only seen in patients with adverse risk disease (138). This should be interpreted with caution as RATIFY was not powered for these subgroup analyses. Nonetheless, this study provides support that in low or even intermediate risk *FLT3-*ITD AML, HCT could potentially be delayed until first relapse or MRD positivity. Of note, in RATIFY, patients that did not receive HCT did receive post-consolidation midostaurin maintenance, an indication that was not approved as discussed above.

More recently, in a phase II study of crenolanib plus chemotherapy followed by crenolanib maintenance in newly diagnosed *FLT3-*mutated AML, 85% of patients achieved CR. Of the 27 patients on trial, 7/27 received consolidation but not HCT; of those, 6/7 remained in long-term remission. OS was similar between patients who underwent HCT *vs* those who did not (55). While the number of patients is small and the ELN risk category not specified, this again raises the question of whether HCT is needed in all patients with *FLT3*-mutated AML.

In a recent position statement by the European Society for Blood and Marrow Transplantation (EBMT), HCT in first CR was recommended for patients with intermediate or adverse risk *FLT3-*ITD AML and could be considered in low risk disease. Furthermore, in the absence of GVHD, post-HCT FLT3 inhibitor maintenance was recommended, ideally on a clinical trial (141). Randomized trials are needed to further clarify these algorithms. Multiple trials of FLT3 inhibitors plus chemotherapy in newly diagnosed AML are actively accruing, and it will be interesting to see the role of HCT in these studies.

#### Given New FLT3-Directed Therapies, Will We Need to Re-Think Risk Stratification?

The current ELN criteria were developed before FLT3 inhibitors were routinely used. Prior to widespread FLT3 inhibitor use, the prognosis for *FLT3-*ITD AML was dismal. Historically, while patients with *FLT3-*ITD responded to induction chemotherapy with similar remission rates as other AML subtypes, patients were more likely to relapse and had inferior OS (7, 142).

In a retrospective analysis of the RATIFY trial, for all enrolled patients, OS differed significantly among ELN risk groups. In all risk groups, midostaurin significantly improved OS, with 5-year OS probabilities for the midostaurin arm of 0.53 and 0.52 for intermediate- and adverse-risk, respectively (138). More recently crenolanib plus chemotherapy in newly diagnosed AML has demonstrated a 3-year OS of 0.76 in adults ≤60 (55) and a 1-year OS of 0.67 in adults 61–75 (56). As a comparison, in at retrospective validation of ELN risk stratification in newly-diagnosed patients receiving conventional chemotherapy, 5-year OS probabilities were 0.36 for *FLT3*-ITD^low^/*NPM1*^WT^ and 0.29 for *FLT3*-ITD^high^/*NPM1*^mutated^ (intermediate risk) and 0.09 for *FLT3-*ITDhigh/*NPM-*WT (adverse risk) (143). While historic comparisons must be interpreted with caution, this may suggest FLT3 inhibitors have shifted the risk associated with *FLT3-*mutated AML.

Given the multiple FLT3-directed therapies both approved and in development, the prognosis of *FLT3-*mutated AML may be changing. Will *FLT3* mutations in AML eventually be analogous to HER2 amplification in breast cancer or *BCR-ABL1* fusions in acute lymphoblastic leukemia? In both of these cases, the development of targeted therapies has dramatically improved the prognosis of a previously poor-risk subtype, and a similar pattern may evolve with *FLT3* as well.

### What Is the Prognostic and Therapeutic Impact of Non-Canonical FLT3 Mutations?

As sequencing technology improves, *FLT3* mutations outside of the ITD and D835 regions have been described. These non-canonical (NC) mutations are frequently in exon 14 of the juxtamembrane (JM) domain, where ITDs occur, or in the KD domain adjacent to D835; however NC mutations in other sites, including the extracellular (EC) domain, have been described as well. Select NC mutations identified in the clinical literature are summarized in **Table 7**.

**Table 7:** Non-Canonical Mutations Identified in Clinical Studies.

Mutation	Exon	Domain	Clinical Activity
D200N	5	EC	Maintained through crenolanib treatment (64)
T227M	6	EC	Confers resistance to sorafenib (68)
K429E	10	EC	Maintained through crenolanib treatment (64)
S451F	11	EC	Pediatric AML (5); Adult AML (4); minimal midostaurin sensitivity (144)
V491L	12	EC	Pediatric AML (5); Adult AML (4)
Y572C	14	JM	High midostaurin sensitivity (144); maintained through crenolanib treatment (64)
E573D/G	14	JM	Pediatric AML (145)
L576R	14	JM	Pediatric AML (145)
T582N	14	JM	Pediatric AML (145)
D586Y	14	JM	Pediatric AML (145)
Y589H	14	JM	Pediatric AML (145)
V592A/G	14	JM	Pediatric AML (5); Adult AML (4); high midostaurin sensitivity (144); clinical sorafenib response (146)
E596K/G	14	JM	Pediatric AML (145)
E598D	14	JM	Adult AML (4); Pediatric AML (5, 145); found after relapse on Giltertininb (62)
Y599C	14	JM	Pediatric AML (145)
D600G	14	JM	Pediatric AML (145)
L601F	14	JM	Mutation maintained through crenolanib treatment (64)
N676D/K	16	KD	Clinical sorafenib response (146), Resistance to midostaurin (147); **r**esistance to quizartinib (148)
G697R	16	KD	Resistance to quizartinib (148)
A833S	20	AL	Eliminated with crenolanib treatment (64)
R834Q	20	AL	High Midostaurin sensitivity (144)
D839Y/G	20	AL	Eliminated with crenolanib treatment (64)
N841K	20	AL	Eliminated with crenolanib treatment (64)
Y842C	20	AL	Eliminated with crenolanib treatment (64)

In the pediatric literature, whole genome sequencing of 799 pediatric AML patients revealed a 7.6% prevalence of NC *FLT3* point mutations and insertions-deletions compared to a 23% total prevalence of all *FLT3* mutations (including *FLT-*ITD and D835). This included 9 JM mutations with activating potential (E598D, E573D/G, L576R, T582N, D586Y, Y589H, E596K/G, Y599C, D600G), many which occurred as co-mutations with *FLT3-*ITD (145). Similarly, in a large study of 1,540 adult AML patients, targeting genetic sequencing identified four NC *FLT3* driver mutation, including two EC (S451F and V491L) and two JM (V592A, E598D) mutations (4).

It is unclear to whether NC mutations confer FLT3 inhibitor susceptibility, resistance, or are simply passenger mutations unimportant to disease biology. In one study of 222 AML patients without *FLT3-ITD* or *-D835* mutations, four NC driver mutations were identified which had variable sensitivity to midostaurin (144). Similarly, in a study of 18 patients treated with crenolanib, pre- and post-treatment sequencing identified small populations of four NC mutations at baseline which were eliminated over the course of treatment (A833S, D839Y/G, N841K, Y842C) (64). In a recent *in vitro* study, gilteritinib was active against multiple NC mutations, including mutations like N676, which are associated with resistance to midostaurin and quizartinib (149). Finally, in a recent case series, two patients with non-*FLT3-*ITD or D835 AML were found to have JM *FLT3-V592G* and KD *FLT3-N676K* mutations, both of which clinically responded to sorafenib (146).

To contrast, maintenance or development of NC mutations have also been observed in conjunction with FLT3 inhibitor resistance. In patients treated with crenolanib, four NC *FLT3* mutations (D200N, K429E, Y572C, L601F) were maintained at time of relapse (64). In an analysis of 40 patients with *FLT3-*mutated AML treatment with gilteritinib on the ADMIRAL trial, six patients had new *FLT3* mutations at time of relapse, five of which were in gatekeeper *F691L* and two at the NC site JM E598D (62).

Many questions remain regarding the nature of NC mutations, including their true prevalence (as they are not detected outside of whole-exome or genome sequencing), prognostic impact, and role in the FLT3 inhibitor susceptibility and resistance.

### Is There a Role for FLT3 Inhibitors in FLT3-WT Disease?

Although only one-quarter of AML patients harbor *FLT3* mutations, the FLT3 receptor is over-expressed on leukemic blast regardless of mutational status (2), and early studies of *FLT3* inhibitors observed blast reduction in patients with *FLT3-*WT disease (19).

Early trials of *FLT3* inhibitors were not limited to patients with *FLT3*-mutated AML, and results from these studies may indicate benefit in targeting *FLT3*-WT. In the RATIFY trial, using an arbitrary AR cut-off of ≥0.7, *post-hoc* analysis noted a similar OS benefit with*FLT3*-ITD^low^, *FLT3*-ITD^high^, and *FLT3-*TKD AML (20). Similarly, in the SORAML trial, EFS benefit and trend toward OS benefit were demonstrated irrespective of *FLT3* mutation status (23, 34).

Given that both midostaurin and sorafenib are multikinase inhibitors, it is possible these results are due to inhibition of alternative kinase-dependent pathways. For example, both sorafenib and midostaurin also inhibit KIT, and *KIT* mutations are seen in 30–46% of core binding factor (CBF) AML and may impact prognosis (150, 151). The use of midostaurin in CBF AML is currently being explored in a phase II study (NCT03686345), and a trial of midostaurin in *c-KIT* or *FLT3*-ITD mutated t(8,21) AML recently completed accrual (NCT01830361).

Efficacy in *FLT3-*WT disease has also been demonstrated in second generation FLT3 inhibitors, which are more specific to the FLT3 receptor. While the initial phase I/II study of gilteritinib demonstrated strongest antileukemic effect in patients with *FLT3-*mutated disease, a 12% ORR was seen in *FLT3-*WT AML (52). Similarly, quizartinib monotherapy demonstrated 54 and 32% ORR in patients with *FLT3-ITD* and *FLT3-*WT disease, respectively (152).

Trials of FLT3 inhibitors in *FLT3*-WT AML are ongoing, including a randomized phase III trial of chemotherapy +/− midostaurin (NCT03512197) and chemotherapy +/− quizartinib (NCT04107727), both in patients with newly-diagnosed *FLT3*-WT AML. In addition, many phase I trials of novel combination therapies, dual FLT3 inhibitors, and biologic agents are not restricted to *FLT3*-mutant disease.

#### How Should We Approach Tumor Heterogeneity in FLT3-Mutant AML?

Increasingly, AML is understood as a heterogenous disease with multiple genetically distinct subclones, dynamically evolving under pressure of therapy. Clonal evolution is particularly relevant in *FLT3-*mutated AML as *FLT3* mutations can be gained with disease progression, and development of a new *FLT3-*ITD is an independent negative prognostic factor (153).

Development of *FLT3*-mutated clones can arise under targeted therapy. In patients with R/R AML treated with venetoclax, analysis of pre- and post-treatment samples demonstrated 4/20 patients developed new *FLT3-*ITD clones following therapy (94). In a larger study of 81 patients treated with frontline venetoclax-based combinations, 5/25 patients showed increased *FLT3-*ITD clonal burden at relapse, two of which had newly acquired *FLT3-*ITD clones (95). Similarly, in patients with *IDH1*-mutated AML treated with ivosidenib, bulk NGS at time of progression identified multiple patients with new *FLT3-*ITD or -TKD mutations not present at therapy initiation (154), and in patients with *IDH2-*mutated AML treated with enasidenib, analysis of paired pre- and post-treatment samples described several cases with increased *FLT3* variant allele frequency at relapse (155).

Complex clonal evolution has also been observed following FLT3 inhibition. In an analysis of patients treated with single agent gilteritinib on the phase III ADMIRAL or phase II CHRYSALIS trial, targeted NGS identified emerging clones with mutations activating RAS/MAPK signaling, including *NRAS* and *KRAS*. Serial single-cell sequencing confirmed early selection for *RAS-*mutant subclones under gilteritinib pressure (63). In an analysis of paired pre- and post-treatment samples of patients treated with quizartinib, *NRAS* development was similarly noted at relapse; furthermore, single cell sequencing confirmed these distinct subclones existed in small populations prior to therapy and expanded under FLT3 inhibition (66).

It is unclear how to best address clonal evolution and associated adaptive resistance in AML. Is the treatment of multiple clones best approached through blunt approaches, such as cytotoxic chemotherapy or broad, triple-therapy combinations, such as HMA/Venetoclax/Gilteritinib? Alternatively, would it be more beneficial to target individual subclones sequentially, perhaps focusing on the highest-risk or fastest-growing subclones first? As genomic technologies such as single cell sequencing become more widely adopted, available genomic information will increase dramatically. Looking forward, innovative bioinformatic and machine learning-based approaches may be employed to rationally treat the complex clonal architecture in *FLT3-*mutated AML.

## Treating *FLT3*-Mutated AML Today

The current standard of care for an adult with newly diagnosed *FLT3-*mutated AML with AR ≥0.05 who is eligible for treatment is induction and consolidation chemotherapy in combination with midostaurin. As *FLT3* AR is not universally reported, if not available the presence of a *FLT3-*ITD or TKD mutation should warrant treatment with a FLT3 inhibitor. This is generally followed by HCT, as the largest survival benefit in the RATIFY trial was observed in patients who underwent HCT in first remission (20), although whether this approach holds in patients with low-AR *FLT3-*ITD, *FLT3-*TKD, or concomitant *NPM1* mutations is debatable.

While not standard of care, use of single-agent maintenance FLT3 inhibitors, either in remission for patients not going to HCT or in post-HCT remission can be considered. This would ideally be done in a clinical trial setting, although off-label use of sorafenib or midostaurin maintenance is routinely practiced in the US (156, 157). We await the results of ongoing clinical trials to help determine in which settings FLT3 inhibitor maintenance is most useful.

**Figure 3:**
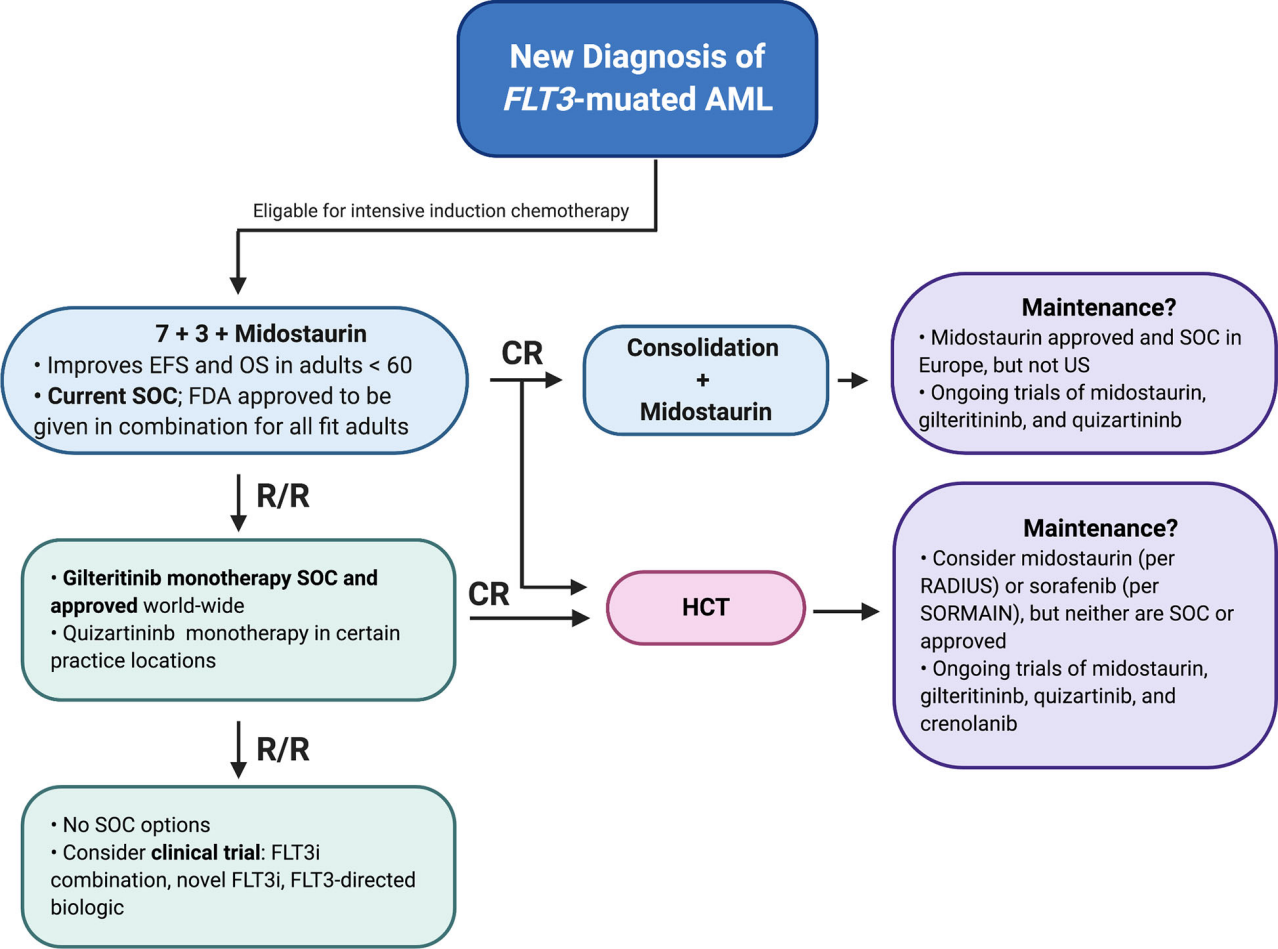
Current Standard of Care for Treating *FLT3-*mutated AML in the Fit Patient.

Should a patient with *FLT3*-ITD AML relapse or a patient with *FLT3-*WT AML develop a new *FLT3*-mutated clone, then single-agent gilteritinib is standard salvage therapy world-wide and single-agent quizartinib could be considered in certain practice locations. If response if achieved, HCT would be recommended in all fit patients. If a patient remains refractory or develops relapse, then multiple clinical trials could be considered, including FLT3 inhibitor combinations, novel FLT3 inhibitors, or FLT3-directed biologics (**Figure 3**). Although both gilteritinib and venetoclax are FDA-approved, and there is promising pre-clinical data on this combination (92), this regimen can be associated with marked myelosuppression and we would not recommend it outside of a clinical trial. Similarly, the triplet combination of HMA/Gilteritinib/Venetoclax should only be used as part of clinical trial until the toxicities associated with this novel combination are better understood.

Finally, special consideration should be given to older adults, who may be intolerant of intensive induction chemotherapy as well as the side effects associated with multikinase inhibitors. As described above, multiple studies are actively investigating FLT3 inhibitors in combination with lower-intensity therapies currently approved as frontline therapy for older adults, including HMAs as well as liposomal cytarabine and anthracycline (CPX-351, Vyxeos).

## Concluding Remarks

While this provides a baseline treatment paradigm, survival remains poor in *FLT3*-mutated AML, and additional treatment options are needed. This includes increasing the diversity of approved FLT3, investigating FLT3 inhibitors in new combinations and treatment settings, and development of novel agents. Given the diversity in FLT3 inhibitor potency, resistance patterns, and toxicity profiles, ideally the oncologist will have a range of FLT3-directed therapies to choose from, similar to TKI selection in chronic myelogenous leukemia, so that a particular FLT3 inhibitor could be matched to an individual patient’s needs.

## Author Contributions

VK and CS both contributed to the writing of this manuscript. All authors contributed to the article and approved the submitted version.

## Conflict of Interest

The authors declare that the research was conducted in the absence of any commercial or financial relationships that could be construed as a potential conflict of interest.
